# The burden of the coronavirus disease 2019 virus infection in Burkina Faso: Results from a World Health Organization UNITY population‐based, age‐stratified sero‐epidemiological investigation

**DOI:** 10.1111/irv.13216

**Published:** 2023-11-17

**Authors:** Samiratou Ouedraogo, Isidore Tiandiogo Traoré, Dramane Kania, Nongodo Firmin Kaboré, Ariane Mamguem Kamga, Hermann Badolo, Mimbouré Yara, Guillaume Sanou, Amariane Koné, Samdapawindé Thérèse Kagoné, Esperance Ouédraogo, Blahima Konaté, Rachel Médah, Nathalie de Rekeneire, Armel Poda, Arnaud Eric Diendere, Boukary Ouédraogo, Oumar Billa, Gilles Paradis, Halidou Tinto, Tienhan Sandrine Dabakuyo‐Yonli

**Affiliations:** ^1^ Observatoire national de la santé de la population (ONSP) Institut National de Santé Publique (INSP) Ouagadougou Burkina Faso; ^2^ Institut National de Santé Publique du Québec (INSPQ) Montreal Quebec Canada; ^3^ The Department of Epidemiology, Biostatistics and Occupational Health, Faculty of Medicine McGill University Montreal Quebec Canada; ^4^ Centre MURAZ, Institut National de Santé Publique Bobo‐Dioulasso Burkina Faso; ^5^ Institut Supérieur des Sciences de la Santé (INSSA) Université Nazi Boni Bobo‐Dioulasso Burkina Faso; ^6^ Epidemiology and Quality of Life Research Unit, INSERM U1231 Georges Francois Leclerc Centre—UNICANCER Dijon France; ^7^ Centre National de Recherche et de Formation sur le Paludisme Institut National de Santé Publique Ouagadougou Burkina Faso; ^8^ Département de médicine, pharmacopée traditionnelle et pharmacie Institut de Recherche en Sciences de la Santé (IRSS)—Centre National de la Recherche Scientifique et Technologique (CNRST) Ouagadougou Burkina Faso; ^9^ Institut des Sciences des Sociétés (INSS)—Centre National de la Recherche Scientifique et Technologique (CNRST) Ouagadougou Burkina Faso; ^10^ Expertise France Paris France; ^11^ Service des maladies infectieuses Centre Hospitalier Universitaire Sourô Sanou Bobo‐Dioulasso Burkina Faso; ^12^ Centre Hospitalier Universitaire de Bogodogo Ouagadougou Burkina Faso; ^13^ Direction des systèmes d'information en santé (DSIS), Ministère de la santé et de l'hygiène publique Ouagadougou Burkina Faso; ^14^ Institut de Recherche en Sciences de la Santé (IRSS)—Unité de Recherche Clinique de Nanoro Centre National de la Recherche Scientifique et Technologique (CNRST) Ouagadougou Burkina Faso

**Keywords:** Burkina Faso, COVID‐19, infectious disease, population‐based seroprevalence, SARS‐CoV‐2, WHO UNITY study

## Abstract

**Background:**

This study aimed to estimate the anti‐SARS‐CoV‐2 antibody seroprevalence in the general population of Bobo‐Dioulasso and Ouagadougou (Burkina Faso).

**Methods:**

We collected from March to April 2021 blood samples from randomly selected residents in both main cities based on the World Health Organization (WHO) sero‐epidemiological investigations protocols and tested them with WANTAI SARS‐CoV‐2 total antibodies enzyme‐linked immunosorbent assay (ELISA) kits intended for qualitative assessment. We also recorded participants' socio‐demographic and clinical characteristics and information on exposure to SARS‐CoV‐2. Data were analysed with descriptive and comparative statistics.

**Results:**

We tested 5240 blood samples collected between 03 March and 16 April 2021. The overall test‐adjusted seroprevalence for SARS‐CoV‐2 antibodies was (67.8% [95% CI 65.9–70.2]) (*N* = 3553/3982). Seroprevalence was highest among participants aged 15–18 years old (74.2% [95% CI 70.5–77.5]) (*N* = 465/627), compared with those aged 10–14 years old (62.6% [95% CI 58.7–66.4]) (*N* = 395/631), or those over 18 (67.6% [95% CI 66.2–69.1]) (*N* = 2693/3982). Approximately 71.0% (601/860) of participants aged 10–18 years old who tested positive for SARS‐CoV‐2 antibodies experienced no clinical COVID‐19 symptoms in the weeks before the survey, compared with 39.3% (1059/2693) among those aged over 18 years old.

**Conclusion:**

This study reports the results of the first known large serological survey in the general population of Burkina Faso. It shows high circulation of SARS‐CoV‐2 in the two cities and a high proportion of asymptomatic adolescents. Further studies are needed to identify the SARS‐CoV‐2 variants and to elucidate the factors protecting some infected individuals from developing clinical COVID‐19.

## 1 INTRODUCTION

On 5 May 2023, the Director‐General of World Health Organization (WHO) announced COVID‐19 emergency over with the decreasing trend in COVID‐19 deaths, the decline in COVID‐19‐related hospitalizations and intensive care unit admissions the high levels of population immunity to SARS‐CoV‐2.^1^ Many governments have adjusted their strategies to reflect the status of the pandemic. But the return to normalcy worldwide, after enforcing severe restrictions to contain COVID‐19, requires continuous efforts to monitor the dynamic of the pandemic and prevent new waves. Indeed, the persistent circulation of SARS‐CoV‐2 and its variants in many countries^2^ call for global monitoring and research and to inform and adjust the COVID‐19 responses. To ensure better preparedness for future pandemic, many countries have embarked on activities to identify the key learnings from the COVID‐19 response to further strengthen health security and health system resilience.^3^ This requires access to quality COVID‐19 epidemiological data^4^ and a deep understanding of the measures developed and implemented by all stakeholders to contribute to control the pandemic.

When exploring the number of cases and deaths officially reported by health authorities, many sub‐Saharan African (SSA) countries appear to have defeated the odds of high morbidity and mortality‐related COVID‐19 and do not constitute a threat to global health security.^5^ Indeed, compared with countries in America, Europe and Asia, the healthcare impact of COVID‐19 seems substantially lower in SSA. For example, in Burkina Faso, 22,056 cumulative confirmed cases of COVID‐19 and 396 cumulative deaths were reported as of 19 July 2023.^6^ However, emerging results from research suggest that weak public health surveillance systems^7^ and low access to healthcare in SSA may have led to shortfalls in the detection and reporting of COVID‐19 cases and deaths. New estimates reveal that the true number of COVID‐19 deaths in Africa may be more than three times higher than that officially reported.^8^, ^9^ This may have resulted in a significant underestimation of the spread of COVID‐19 in the region.^10^, ^11^ Indeed, several seroprevalence studies using non‐standardized methodology have reported conflicting findings regarding the circulation of SARS‐CoV‐2 on the continent, with estimates of seroprevalence ranging from 0% to 59%, depending on the population studied, the sampling strategy, the sample size, the serological test used and the time of the survey.^12^, ^13^ High‐quality, large‐scale seroprevalence studies are needed to more accurately estimate the true burden of COVID‐19 and to understand the dynamics and nature of immune responses to SARS‐CoV‐2 infection in the African epidemiological context.^14^

The WHO developed early investigation protocols for SARS‐CoV‐2 antibodies seroprevalence in the general population and in healthcare settings, branded as the ‘UNITY studies’.^15^ These protocols have been implemented in all WHO regions and half of all Humanitarian Response Plan countries, including Burkina Faso. They may generate standardized and robust local seroprevalence data, as well as key epidemiological, virological and clinical parameters that will improve our understanding of the important characteristics of the SARS‐CoV‐2 infection.^14^ This could also provide insight to inform the design of effective COVID‐19 control programmes and potential similar pandemics, especially in the context of scarce resources, limited access and acceptance of COVID‐19 vaccines, as is the case in Burkina Faso.^16^

The ANRS‐CoV‐13 ‘EMULCOVID’ research group reports here the results of the first known large SARS‐CoV‐2 seroprevalence study performed in the general population of Burkina Faso's two main cities (Ouagadougou and Bobo‐Dioulasso) as part of the WHO ‘UNITY studies’.^15^

## 2 METHODS

### 2.1 Study design and setting

This was a cross‐sectional, population‐based, age‐stratified sero‐epidemiological investigation of COVID‐19 viral infection conducted in the two major cities of Burkina Faso, namely, Ouagadougou, the capital and largest city, and Bobo‐Dioulasso, the second largest city. Burkina Faso is a landlocked country in the Sahel of West Africa and has an estimated population of over 20 million inhabitants.^16^ According to a recent general population housing survey, the cities of Ouagadougou and Bobo‐Dioulasso together account for more than 62% of the urban population, with 2.5 million and 903,000 inhabitants, respectively.^17^ Since the beginning of the pandemic in March 2020, most data on COVID‐19 in BF have been reported from these two cities.

**FIGURE 1:**
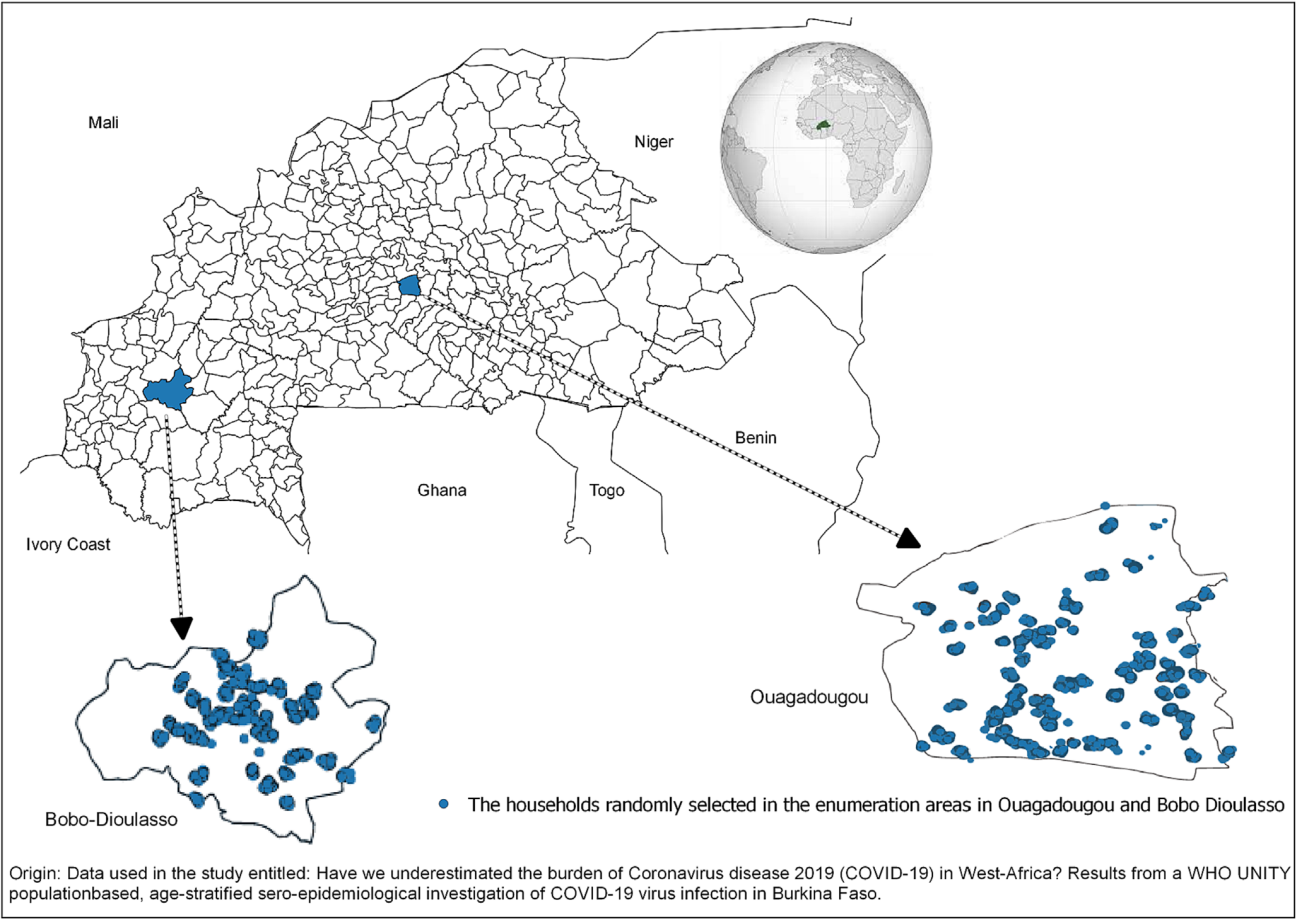
SARS‐CoV‐2 seroprevalence sampling collection sites in Ouagadougou and Bobo‐Dioulasso (Burkina Faso, March 2021).

Ouagadougou is in the centre of the country and had 12 boroughs as of 2017 and 1276 enumeration areas (EAs) (Figure 1). The city of Bobo‐Dioulasso, located in the southwest of the country, had seven boroughs and 536 EAs (Figure 1). In both cities, each EA had an average of 220 households and each household comprised an average of 4.8 people.^17^ Figure 1 presents the EAs of Ouagadougou and Bobo‐Dioulasso where the data were collected.

### 2.2 Participants

We use the sample size calculator available on OpenEpi^18^ to estimate the sample size considering an expected SARS‐CoV‐2 seroprevalence of 10% (based on data reported at the study period),^19^ an accuracy of ± 3%, an alpha risk = 5% and no design effect (Deff = 1). An estimated 384 persons in each gender and age stratum was required, and this number was rounded to 452 to account for approximately 85% usable data. Full details of the study sampling method have been described elsewhere.^20^ We used a two‐stage sampling strategy: We sampled EA and then long‐term residents aged 10 years and older from each sampled household, stratifying by age group (10–18 years, 19–50 years, 60 years and older) and sex (male, female). We invited those selected to provide a blood sample and complete a questionnaire. In some households, more than one resident was sampled (up to five maximum), but they were from different age and sex groups.

### 2.3 Data sources/measurement

Qualified, accredited field staff collected blood and administered a questionnaire.

In total, 4 mL of venous blood was drawn into a 5 mL EDTA tube, triple‐packed in an isotherm container with an icebox and transferred to the either of the main reference laboratories of Burkina Faso within 6 h, namely, the Laboratory of the National Center for Malaria Research and Training in Ouagadougou or the Laboratory of the MURAZ Centre in Bobo‐Dioulasso. Plasma was centrifuged and aliquoted within 12 h of collection.

#### 2.3.1 SARS‐CoV‐2 seroprevalence testing

We measured SARS‐CoV‐2 antibodies in the collected blood samples using the WANTAI SARS‐CoV‐2 Ab enzyme‐linked immunosorbent assay (ELISA), an ELISA intended for qualitative detection of total antibodies (including IgG and IgM) to SARS‐CoV‐2 in human serum and acid citrate dextrose (ACD) plasma. The WANTAI SARS‐CoV‐2 Ab ELISA is intended for use as an aid in identifying individuals with an adaptive immune response to SARS‐CoV‐2, indicating recent or prior infection. The WANTAI SARS‐CoV‐2 Ab ELISA cannot discriminate IgG and IgM.

The test results of the study participants were the main outcomes of the study. Data were recorded in an electronic case report form, along with the questionnaire data.

#### 2.3.2 Questionnaire data

Each study participant completed a questionnaire as previously described by Traoré et al.^20^ to collect socio‐demographic characteristics (age, profession, marital status, level of education and socio‐professional category), prior medical history (history of cardiovascular and thrombo‐embolic disease [arterial hypertension, rhythm disorders, cardiomyopathy, deep venous thrombosis and/or pulmonary embolism]), history of metabolic diseases (diabetes mellitus and kidney failure), history of pulmonary disease (tuberculosis, asthma and chronic obstructive pulmonary disease [COPD]) and other relevant medical history. The questionnaire also asked about lifestyle habits: tobacco and alcohol consumption, clinical signs of COVID‐19 (fever [temperature ≥38°C], cough, dyspnoea, etc.) and exposure to COVID‐19 up to 14 days prior to data collection (any contact with a confirmed case of COVID‐19).

### 2.4 Analyses

We described the socio‐economic characteristics of the study population. Quantitative variables were described as mean ± standard deviation (SD) and qualitative variables as number and percentage. The crude and adjusted SARS‐CoV‐2 seroprevalence were estimated with 95% confidence intervals in adolescents, overall and by age group and sex. We compared the characteristics of SARS‐CoV‐2 positive versus negative participants. We described COVID‐19 symptoms in the participants who tested positive for SARS‐CoV‐2 antibodies using numbers and percentage. All analyses were conducted separately for adults and adolescents and were presented separately by age and sex groups. SAS version 9.4 (SAS Institute Inc., Cary, NC, USA) was used to perform all analyses.

People within the same household and EA are more likely to resemble each other than people in other areas and are more likely to share risk factors for infection (e.g., genetic, environmental and socio‐economic). Infectious disease tends to cluster in time and space. This dependence in the data was therefore accounted for in the statistical analysis because each person provides less information than if their response was independent of other people in the same area. To account for clustering at the household and EA level, all analyses were adjusted per EA using standard methods to obtain point estimates of seroprevalence with ‘robust’ standard errors that are calculated based on the observed variability in seroprevalence among clusters. The adjustment was done using SAS (PROC SURVEYFREQ).

## 3 RESULTS

### 3.1 Characteristics of the study population

The population had a good acceptance of the study despite the need for blood sampling. Only 29% (2138/7402) of the residents sampled either refused or were absent from home (repeatedly absent) during the data collection period.

A total of 5264 residents were sampled from 03 March to 16 April 2021. After blood sample collection and testing, data from 5240 individuals were validated and analysed: 67.6% (*N* = 3540) in Ouagadougou and 32.4% (*N* = 1700) in Bobo‐Dioulasso. Data from 12 individuals were duplicated with conflicting seroprevalence results and were excluded from the analysis.

In total, 32.1% of participants were aged 60 years and older (*N* = 1426), and 51.7% (*N* = 2000) were female. More than 26% (*N* = 1382) of the study participants had received zero schooling.

The most frequent comorbidities reported were in patients with high blood pressure 13.8% (*N* = 722), chronic neurological or neuromuscular disease 4.5% (*N* = 238) or diabetes 3.1% (*N* = 162). More than 89% (*N* = 4686) of participants had received the Bacille Calmette–Guérin (BCG) vaccination against tuberculosis.

The percentage of current smokers within the study population was 5.7% (*N* = 299), and 2.7% (*N* = 144) of the study population had received long‐term corticosteroid treatment. Prior to the study, 2.2% (*N* = 115) of the study population had nasopharyngeal swabs to test for COVID‐19 as part of the national response strategy, 7.3% (*N* = 8/115) of which were positive.

Overall, 52.9% (*N* = 2770) of all study participants reported having had at least one clinical symptom suggestive of COVID‐19 during the 2 weeks prior to the data collection. Students represented 15% of SARS‐CoV‐2 positive adults compared with 12.1% among those who tested negative for SARS‐CoV‐2 antibodies.

The full characteristics of the study participants are presented in Table 1.

**TABLE 1:** Characteristics of the study population tested for SARS‐CoV‐2 in Ouagadougou and Bobo‐Dioulasso (Burkina Faso, March 2021).

Variables	10–18 years (*N* = 1258)	19–59 years men (*N* = 1279)	19–59 years women (*N* = 1277)	60 years or over (*N* = 1426)
	*N* (%)	*N* (%)	*N* (%)	*N* (%)
Town				
Bobo‐Dioulasso	409 (32.5)	413 (32.3)	413 (32.3)	465 (32.6)
Ouagadougou	849 (67.5)	866 (67.7)	864 (67.7)	961 (67.4)
Sex				
Female	710 (56.4)	–	–	723 (50.7)
Male	548 (43.6)	–	–	703 (49.3)
Age group (years)				
10–14	631 (50.2)	–	–	–
15–18	627 (49.8)	–	–	–
Education level				
No school	79 (6.6)	127 (9.9)	385 (30.1)	791 (55.5)
Basic literacy	22 (1.8)	69 (5.4)	62 (4.9)	132 (9.2)
Junior high school	464 (38.5)	223 (17.4)	294 (23.0)	260 (18.2)
Senior high school	635 (52.7)	566 (44.3)	435 (34.1)	185 (13.0)
University	5 (0.4)	294 (23.0)	101 (7.9)	58 (4.1)
Unknown	53	–	–	–
Marital status				
Single	–	647 (50.7)	304 (23.8)	15 (1.0)
Living martially	–	32 (2.5)	28 (2.2)	1 (0.1)
Married	–	585 (45.8)	874 (68.5)	905 (63.5)
Separated/divorced	–	9 (0.7)	12 (0.9)	15 (1.0)
Widowed	–	4 (0.3)	58 (4.6)	489 (34.4)
Unknown	–	2	1	1
Profession				
Civil servant	–	78 (6.1)	32 (2.5)	13 (0.9)
Private‐sector worker	–	123 (9.6)	39 (3.1)	26 (1.8)
Tradesman	–	187 (14.6)	264 (20.7)	161 (11.4)
Volunteer	–	32 (2.5)	22 (1.7)	10 (0.7)
Student	–	369 (28.9)	184 (14.4)	4 (0.3)
Homemaker/housewife	–	6 (0.5)	550 (43.1)	471 (33.3)
Informal other than trade	–	245 (19.2)	110 (8.6)	92 (6.5)
Retired	–	13 (1.0)	5 (0.4)	393 (27.8)
Jobseeker	–	26 (2.0)	15 (1.2)	33 (2.3)
Invalidity	–	5 (0.4)	4 (0.3)	104 (7.3)
Other	–	194 (15.2)	51 (4.0)	109 (7.7)
Unknown	–	1	1	10
BMI (kg/m^2^)^a^				
<18.5	–	92 (7.2)	66 (5.2)	134 (9.4)
18.5–24.9	–	804 (62.9)	515 (40.3)	698 (49.0)
25–29.9	–	275 (21.5)	355 (27.8)	364 (25.5)
≥30	–	108 (8.4)	341 (26.7)	230 (16.1)
Tobacco				
Current smoker	8 (0.7)	239 (18.7)	0 (0)	52 (3.7)
Former smoker	0 (0)	135 (10.6)	0 (0)	114 (8.0)
Never smoked	1194 (99.3)	905 (70.7)	1274 (100)	1258 (88.3)
Unknown	56	0	3	2
Alcohol				
No	1245 (99.0)	1001 (78.3)	1146 (89.7)	1251 (87.7)
Yes—misuse^b^	13 (1.0)	241 (18.8)	123 (9.6)	159 (11.2)
Yes—dependence^c^	0 (0)	37 (2.9)	8 (0.6)	16 (1.1)
Medical history				
Diabetes	1 (0.1)	15 (1.3)	30 (2.6)	117 (8.9)
Hypertension	–	51 (4.2)	132 (10.8)	539 (39.2)
Chronic liver disease	1 (0.1)	7 (0.6)	11 (0.9)	2 (0.2)
Chronic heart disease	5 (0.4)	25 (2.0)	40 (3.2)	81 (5.8)
Chronic kidney disease	–	9 (0.7)	20 (1.6)	55 (4.0)
Chronic neurological or neuromuscular disease	2 (0.2)	31 (2.5)	35 (2.8)	170 (12.1)
Chronic rheumatological disease	3 (0.3)	10 (0.8)	37 (2.9)	81 (5.7)
Chronic respiratory disease	15 (1.3)	29 (2.3)	55 (4.3)	39 (2.8)
HIV	–	2 (0.2)	9 (0.7)	3 (0.2)
Tuberculosis	–	0 (0)	0 (0)	6 (0.4)
Malignant tumour	–	0 (0)	1 (0.1)	3 (0.2)
Other	11 (0.9)	82 (6.6)	95 (7.7)	143 (10.4)
BCG vaccine				
Yes	1179 (98.0)	1215 (95.1)	1219 (95.7)	1073 (75.3)
No	24 (2.0)	62 (4.9)	55 (4.3)	351 (24.7)
Unknown	55	2	3	2
Exposure to COVID‐19				
Visiting COVID‐19 patients	3 (0.3)	18 (1.4)	8 (0.6)	10 (0.7)
Working with COVID‐19 patients	1 (0.1)	30 (2.6)	8 (0.7)	6 (0.4)
Contact (<1 m) of a COVID patient	4 (0.3)	29 (2.6)	18 (1.6)	16 (1.2)
Sharing the same environment with a COVID patient	4 (0.3)	36 (3.2)	22 (1.9)	17 (1.3)
Travel with a COVID patient	3 (0.3)	8 (0.7)	5 (0.4)	1 (0.1)
Providing direct care to a COVID patient	–	8 (0.6)	6 (0.5)	4 (0.3)
Have you ever done a nasopharyngeal swabs test for COVID‐19?				
Yes	5 (0.4)	47 (3.7)	29 (2.3)	34 (2.4)
No	1224 (99.6)	1229 (96.3)	1240 (97.7)	1387 (97.6)
Unknown	29	16		
Long‐term corticosteroid therapy (>10 days of treatment)?				
Yes	15 (1.3)	25 (2.0)	67 (5.3)	37 (2.6)
No	1189 (98.7)	1251(98.0)	1210 (94.7)	1380 (97.4)
Unknown	54	3	0	9
Had at least one COVID‐19 symptom^d^	365 (29.7)	711 (55.6)	761 (59.6)	933 (65.4)

Abbreviations: BMI, body mass index; HIV, human immunodeficiency virus.

^a^ For BMI, height and weight were self‐reported.

^b^ Misuse: AUDIT‐c score ≥3 in women or ≥4 in men.

^c^ Dependence: AUDIT‐c score ≥10.

^d^ Participant who reported at least one common COVID‐19 symptoms.

### 3.2 Outcome data

#### 3.2.1 Seroprevalence of SARS‐CoV‐2 antibodies

Overall, the seroprevalence of SARS‐CoV‐2 antibodies was 67.8% (95% CI 65.9–70.2) (*N* = 3553): 68.3% (*N* = 2419) in Ouagadougou and 66.7% (95% CI 63.8–69.5) (*N* = 1134) in Bobo‐Dioulasso (Figure 1). Table 2a,b presents the crude and adjusted seroprevalence of SARS‐CoV‐2 antibodies by sex and age groups.

**TABLE 2:** Crude and adjusted seroprevalence estimates by age group in Ouagadougou and Bobo‐Dioulasso (Burkina Faso, March 2021).

a: Crude and ZD‐adjusted^a^ seroprevalence estimates for anti‐SARS‐CoV‐2 antibodies in adults (aged >18 years)				
Variables	All	SARS‐CoV‐2 seropositive *N* (%)	Seroprevalence (95% confidence interval)	
			Crude	ZD‐adjusted^a^
Overall	3982	2693 (67.6)	67.6 (66.2–69.1)	67.6 (65.9–69.3)
Sex				
Female	2000	1351 (67.5)	67.5 (65.5–69.6)	67.5 (65.2–69.8)
Male	1982	1342 (67.7)	67.7 (65.6–69.8)	67.7 (65.5–69.9)
Age groups				
18–59 years old	2556	1725 (67.5)	67.5 (65.6–69.3)	67.5 (65.5–69.4)
≥60 years old	1426	968 (67.9)	67.9 (65.4–70.3)	67.9 (65.2–70.5)
Age and sex				
19–59‐year‐old men	1279	867 (67.8)	67.8 (65.2–70.3)	67.8 (65.1–70.3)
19–59‐year‐old women	1277	858 (67.2)	67.2 (64.5–69.8)	67.2 (64.3–70.0)
60 years old or over	1426	968 (67.9)	67.9 (65.4–70.3)	67.9 (65.2–70.5)
City				
Bobo‐Dioulasso	1291	861 (66.7)	66.7 (64.1–69.3)	66.7 (63.8–69.5)
Ouagadougou	2691	1832 (68.1)	68.1 (66.3–69.8)	68.1 (65.9–70.2)
Symptoms				
Yes	2405	1634 (67.9)	67.9 (66.0–69.8)	67.9 (66.0–69.8)
No	1577	1059 (67.1)	67.1 (64.8–69.5)	67.1 (64.1–70.1)

b: Crude and ZD‐adjusted^a^ seroprevalence estimates for anti‐SARS‐CoV‐2 antibodies in young participants (aged 10–18 years)				
Variables	All	SARS‐CoV‐2 seropositive *N* (%)	Seroprevalence (95% confidence interval)	
			Crude	ZD‐adjusted^a^
Overall	1258	860 (68.4)	68.4 (65.7–70.9)	68.4 (65.4–71.2)
Sex				
Female	710	470 (66.2)	66.2 (62.6–69.7)	66.2 (62.6–69.7)
Male	548	390 (71.2)	71.2 (67.2–74.9)	71.2 (66.6–75.4)
Age groups				
10–14 years old	631	395 (62.6)	62.6 (58.7–66.4)	62.6 (58.4–66.7)
15–18 years old	627	465 (74.2)	74.2 (70.5–77.5)	74.2 (70.5–77.6)
Age and sex				
Boys 10–14 years old	308	204 (66.2)	66.2 (60.6–71.5)	66.2 (59.9–72.2)
Girls 10–14 years old	323	191 (59.1)	59.1 (53.5–64.5)	59.1 (53.5–64.6)
Boys 15–18 years old	240	186 (77.5)	77.5 (71.7–82.6)	77.5 (71.5–82.8)
Girls 15–18 years old	387	279 (72.1)	72.1 (67.3–76.5)	72.1 (67.3–76.5)
City				
Bobo‐Dioulasso	409	273 (66.7)	66.7 (61.9–71.3)	66.7 (61.3–71.9)
Ouagadougou	849	587 (69.1)	69.1 (65.9–72.2)	69.1 (65.6–72.5)
Symptoms				
Yes	365	246 (67.4)	67.4 (62.3–72.2)	67.4 (62.3–72.2)
No	865	601 (69.5)	69.5 (66.3–72.5)	69.5 (65.9–72.9)

^a^ The ZD‐adjusted uses standard methods to obtain point estimates of seroprevalence but with ‘robust’ standard errors that are calculated based on the observed variability in seroprevalence among clusters.

Participants aged 60 years or more had a seroprevalence of SARS‐CoV‐2 antibodies comparable with the overall study population, at 67.9% (95% CI 65.2–70.5). Seroprevalence was 62.6% (95% CI 58.4–66.7) among those aged 10–14 years, 74.2% (95% CI 70.5–77.6) in participants aged 15–18 years and 67.6% (95% CI 65.9–69.3) among those 18 years and older.

The seroprevalence was highest among boys: 66.2% (95% CI (59.9–72.2) in boys aged 10–14 years old and 77.5% (95% CI 71.7–82.8) in those aged 15–18 years old. In girls, it was 59.1% (95% CI 53.5–64.6) and 72.1% (95% CI 67.3–76.5), respectively.

#### 3.2.2 Comparison of characteristics according to SARS‐CoV‐2 antibody status (Table 3a,b)

**TABLE 3:** Comparison of the characteristics between adult study participants who tested positive for SARS‐CoV‐2 and those who tested negative in Ouagadougou and Bobo‐Dioulasso (Burkina Faso, March 2021).

a: Comparison of the characteristics between adult study participants aged 18+ years old who tested positive for SARS‐CoV‐2 and those who tested negative in Ouagadougou and Bobo‐Dioulasso (Burkina Faso, March 2021)			
Variables	Positive (*N* = 2693)	Negative (*N* = 1289)	*p* value^a^
	*N* (%)	*N* (%)	
City			0.3815
Bobo‐Dioulasso (*N* = 1291)	861 (32.0)	430 (33.4)	
Ouagadougou (*N* = 2691)	1832 (68.0)	859 (66.6)	
Sex			0.9144
Female (*N* = 2000)	1351 (50.2)	649 (50.4)	
Male (*N* = 1982)	1342 (49.8)	640 (49.6)	
Age groups			0.7989
<60 years old (*N* = 2556)	1725 (64.0)	831 (64.5)	
≥60 years old (*N* = 1426)	968 (36.0)	458 (35.5)	
Education level			0.6469
No school (*N* = 1303)	888 (33.0)	415 (32.2)	
Basic literacy (*N* = 263)	171 (6.3)	92 (7.1)	
Primary (*N* = 777)	518 (19.2)	259 (20.1)	
Secondary (*N* = 1186)	799 (29.7)	387 (30.0)	
University (*N* = 453)	317 (11.8)	136 (10.6)	
Marital status			0.0467
Single (*N* = 966)	689 (25.6)	277 (21.5)	
Living maritally (*N* = 61)	39 (1.5)	22 (1.7)	
Married (*N* = 2364)	1568 (58.3)	796 (61.7)	
Separated/divorced (*N* = 36)	21 (0.8)	15 (1.2)	
Widowed (*N* = 551)	372 (13.8)	179 (13.9)	
Profession			0.0405
Civil servant (*N* = 123)	76 (2.8)	47 (3.6)	
Private‐sector worker (*N* = 188)	138 (5.1)	50 (3.9)	
Tradesman (*N* = 612)	413 (15.4)	199 (15.5)	
Volunteer (*N* = 64)	41 (1.5)	23 (1.8)	
Student (*N* = 557)	401 (15.0)	156 (12.1)	
Homemaker/housewife (*N* = 1027)	694 (25.9)	333 (25.9)	
Informal other than trade (*N* = 447)	301 (11.2)	146 (11.3)	
Retired (*N* = 411)	283 (10.5)	128 (9.9)	
Jobseeker (*N* = 74)	42 (1.6)	32 (2.5)	
Invalidity (*N* = 113)	72 (2.7)	41 (3.2)	
Other (*N* = 354)	222 (8.3)	132 (10.3)	
BMI (kg/m^2^)			0.1151
<18.5 (*N* = 292)	182 (6.8)	110 (8.5)	
18.5 ≤ BMI < 25 (*N* = 2017)	1360 (50.5)	657 (51.0)	
25 ≤ BMI < 30 (*N* = 994)	674 (25.0)	320 (24.8)	
≥30 (*N* = 477)	477 (17.7)	202 (15.7)	
Tobacco			0.0413
Current smoker (*N* = 291)	180 (6.7)	111 (8.6)	
Former smoker (*N* = 249)	161 (6.0)	68 (6.9)	
Never smoked (*N* = 3437)	2351 (87.3)	1086 (84.5)	
Alcohol			0.3517
No (*N* = 3398)	2313 (85.9)	1085 (84.2)	
Yes—misuse (*N* = 523)	341 (12.7)	182 (14.1)	
Yes—dependence (*N* = 61)	39 (1.4)	22 (1.7)	
Long‐term corticosteroid therapy (>10 days of treatment)?			0.5898
Yes (*N* = 129)	90 (3.4)	39 (3.0)	
No (*N* = 3841)	2593 (96.6)	1248 (97.0)	
Symptoms			0.6028
Yes (*N* = 2405)	1634 (60.7)	771 (59.8)	
No (*N* = 1577)	1059 (39.3)	518 (40.2)	

b: Comparison of the characteristics between young study participants aged 10–18 years old who tested positive for SARS‐CoV‐2 and those who tested negative in Ouagadougou and Bobo‐Dioulasso (Burkina Faso, March 2021)			
Variables	Positive (*N* = 860)	Negative (*N* = 398)	*p* value^a^
	*N* (%)	*N* (%)	
City			0.3928
Ouagadougou (*N* = 849)	587 (68.3)	262 (65.8)	
Bobo‐Dioulasso (*N* = 409)	273 (31.7)	136 (34.2)	
Sex			0.0602
Female (*N* = 710)	470 (54.6)	240 (60.3)	
Male (*N* = 548)	390 (45.4)	158 (39.7)	
Age			<0.0001
10–14 years (*N* = 631)	395 (45.9)	236 (59.3)	
15–18 years (*N* = 627)	465 (54.1)	162 (40.7)	
Education level			0.0006
No school (*N* = 79)	44 (5.3)	35 (9.3)	
Basic literacy (*N* = 22)	17 (2.1)	5 (1.3)	
Junior high school (*N* = 464)	298 (35.9)	166 (44.1)	
Senior high school (*N* = 635)	465 (56.1)	170 (45.2)	
University (*N* = 5)	5 (0.6)	0 (0)	
Unknown	31	22	
Profession			0.9641^a^
Tradesman (*N* = 2)	1 (0.3)	1 (0.5)	
Student (*N* = 565)	360 (94.2)	205 (94.1)	
Homemaker/housewife (*N* = 9)	5 (1.3)	4 (1.8)	
Informal other than trade (*N* = 10)	6 (1.6)	4 (1.8)	
Invalidity (*N* = 1)	1 (0.3)	0 (0)	
Other (*N* = 13)	9 (2.4)	4 (1.8)	
Unknown	478	180	
Symptoms			0.4712
Yes (*N* = 365)	246 (29.0)	119 (31.1)	
No (*N* = 865)	601 (71.0)	264 (68.9)	
Unknown	13	15	
Alcohol			0.5629^a^
No (*N* = 1245)	852 (99.1)	393 (98.7)	
Yes—misuse (*N* = 13)	8 (0.9)	5 (1.3)	
Tobacco			0.4472^a^
Current smoker (*N* = 8)	7 (0.9)	1 (0.3)	
Never smoked (*N* = 1194)	821 (99.1)	373 (99.7)	
Unknown (*N* = 56)			
Long‐term corticosteroid therapy (>10 days of treatment)?			1.000^a^
Yes (*N* = 15)	11 (1.3)	4 (1.1)	
No (*N* = 1189)	818 (98.7)	371 (98.9)	
Unknown	31	23	

^a^ The *p* value was calculated using chi‐square, Freeman–Halton or Fischer tests.

Adults who tested positive for SARS‐CoV‐2 antibodies had similar characteristics to those who tested negative, except that they were more likely to be single (25.6% [*N* = 689] among adults who tested positive for SARS‐CoV‐2 antibodies, compared with 21.5% [*N* = 277] among those who tested negative). Students represented 15.0% (*N* = 401) of SARS‐CoV‐2 antibodies positive adults compared with 12.1% (*N* = 156) among those who tested negative for SARS‐CoV‐2 antibodies. Those who tested positive were also less likely to be current smokers (6.7%, *N* = 180), in comparison with 8.6% (*N* = 111) who tested negative.

Adolescents 15–18 years old were most likely to have positive SARS‐CoV‐2 antibodies, at 54.1% (*N* = 465) compared with the 40.7% (*N* = 162) who tested negative for SARS‐CoV‐2 antibodies. They were also more likely to be in secondary school (56.1% (*N* = 465), compared with 45.2% (*N* = 170) in those who tested negative).

Lifestyle, clinical history of disease and current treatment plans were similar among those who tested positive and negative for SARS‐CoV‐2 antibodies. Among participants who tested positive for SARS‐CoV‐2 antibodies, more than 39% had no current clinical symptoms related to COVID‐19.

#### 3.2.3 History of COVID‐19 symptoms in participants who tested positive for SARS‐CoV‐2 antibodies, by age group (Figure 2)

**FIGURE 2:**
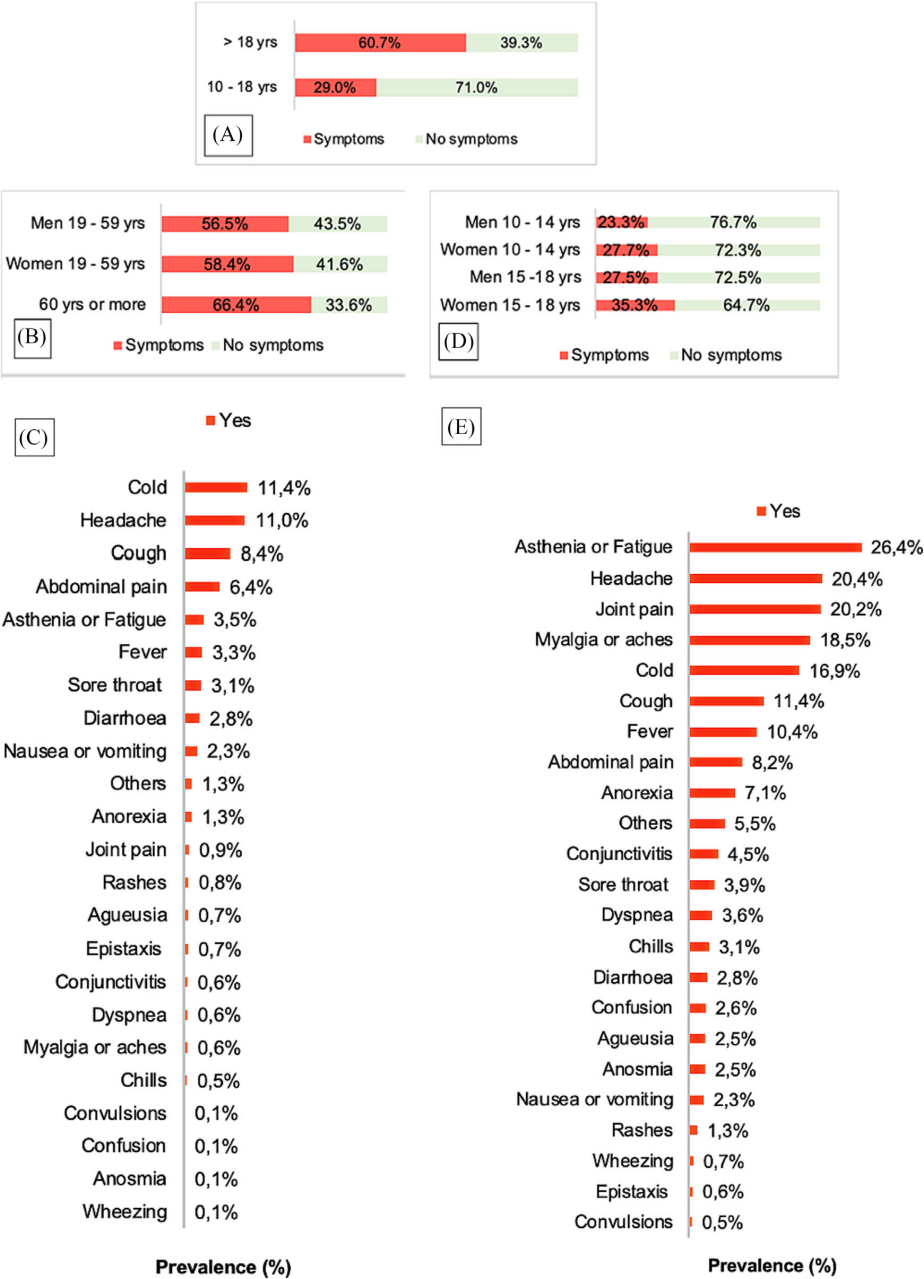
Description of symptoms suggestive of COVID‐19 in study participants who tested positive for SARS‐CoV‐2 antibodies by age group in Ouagadougou and Bobo‐Dioulasso (Burkina Faso, March 2021). (A) Prevalence of symptoms in participants who tested positive for anti‐SARS‐CoV‐2 antibodies according to age. (B) Prevalence of symptoms in participants who tested positive for anti‐SARS‐CoV‐2 antibodies according to age and sex in participants aged over 18 years old. (C) Description of symptoms suggestive of COVID‐19 in participants aged over 18 years old, (D) Prevalence of symptoms in participants who tested positive for anti‐SARS‐CoV‐2 antibodies according to age and sex in participants aged 10–18 years old. (E) Description of symptoms suggestive of COVID‐19 in participants aged 10–18 years old.

Adolescents who tested positive for SARS‐CoV‐2 antibodies reported fewer COVID‐19 clinical symptoms in the 2 weeks prior to the survey than adults. In fact, 71.0% of participants aged 10–18 years old who tested positive for SARS‐CoV‐2 antibodies reported experiencing no COVID‐19 clinical symptoms in the weeks before the survey, compared with the 39.3% symptomless among those aged 18 years and over. The most common symptoms reported primarily by adults who tested positive for SARS‐CoV‐2 antibodies were fatigue, headache, joint pain and myalgia. Adolescents primarily reported experiencing chills, headache, cough and abdominal pain.

## 4 DISCUSSION

Our study highlighted a high seroprevalence of total SARS‐CoV‐2 antibodies in the general population of adolescent and adult residents in the two major cities of Burkina Faso. To the best of our knowledge, as of 13 June 2022, these estimates are among the highest seroprevalence data reported from studies performed in the general population worldwide. Several studies conducted at the earliest stage of the pandemic that used different samples or analysis protocols reported lower SARS‐CoV‐2 antibodies seroprevalence in various groups of the population.^21^, ^22^, ^23^, ^24^ For instance, Nwosu et al. reported a test‐adjusted seroprevalence of 29.2% for anti‐SARS‐CoV‐2 IgG antibodies in a random sample of residents of a health district in Yaoundé, Cameroon, tested from 14 October to 26 November 2020.^23^ Nkuba Ndaye et al. found a seropositivity rate between 13% and 36% in a cross‐sectional serological survey of staff working in healthcare facilities in Kinshasa, the capital of the Democratic Republic of the Congo.^25^ Using data collected between February and June 2021, Struck et al. reported SARS‐CoV‐2 antibodies seroprevalence adjusted for test performance and population characteristics of 37.4% in Bobo‐Dioulasso and 41.5% in Ouagadougou.^26^ However, their study sample was smaller than ours, which resulted in wider confidence intervals between their estimates.

Like our study, other research conducted in general populations using samples collected at least 1 year after the WHO declared COVID‐19 to be a public health emergency of international concern has also reported high anti‐SARS‐CoV‐2 antibody seroprevalence rates. A cross‐sectional population‐based study performed by Moreira‐Soto et al. in San Martin, a rural area of Peru, reported an overall seroprevalence of SARS‐CoV‐2 IgG antibodies of 59.0% (95% CI: 55% to 63%).^27^ In a systematic review and meta‐analysis of standardized population‐based seroprevalence studies, Bergeri et al. reported the global epidemiology of SARS‐CoV‐2 infection from January 2020 to December 2021.^28^ They show that in the first half of 2021, SARS‐CoV‐2 reached 70.1% in Africa due to infection mainly. In Mali, Sagara et al. reported an anti‐SARS‐CoV‐2 antibody seroprevalence of 73.4% in the urban community of Sotuba, 53.2% in the rural town of Bancoumana and 37.1% in the rural village of Donéguébougou.^29^

In Burkina Faso, Peru and Mali, the low number of cases officially reported by health authorities may not reflect the reality of the disease's epidemiological pattern. Indeed, the high anti‐SARS‐CoV‐2 antibody seroprevalence rate reported in these areas supports the fact that PCR‐confirmed case counts are biased proxies for the true attack rate of SARS‐CoV‐2 and may underestimate the true burden of COVID‐19.^23^ This can be explained by several factors, including the weakness of the health system and poor laboratory diagnostic capacity.^7^, ^30^ Although the situation has improved during the COVID‐19 pandemic,^31^ the improvements have not bridged the gap in laboratory diagnostic capacity. Moreover, the concept of disease and health‐seeking behaviour in these countries, on top of the fear of being infected by COVID‐19 in healthcare facilities, reduces the likelihood that some citizens will request care when presented with COVID‐19 symptoms.^32^ Moreover, because some COVID‐19 symptoms are similar to those of existing endemic diseases, such as malaria and dengue, some patients may defer to usual self‐medication.^33^ This behaviour is exacerbated by the stigma and rejection faced by COVID‐19 patients and their families at the beginning of the pandemic.^34^

This study was conducted in the most densely populated cities of Burkina Faso, in EAs and households with different socio‐economic conditions. Most of these households lacked adequate sanitation facilities and basic infrastructures and faced challenges in applying prevention measures, such as social distancing and good hygiene practices. This context may have facilitated wide circulation and high transmission of SARS‐CoV‐2.^26^ Yet, there were minor differences in seroprevalence estimates after adjustment because to the low variability in seroprevalence among clusters. This may be due to the multiple control measures implemented at community level to prevent the spread of the SARS‐CoV‐2.

The young population age structure in Burkina Faso may also have contributed to the low case‐fatality rate when compared with the situation in Europe and North America. About 80% of the population in Burkina Faso is under 35 years of age.^17^ Younger age is thought to be generally protective against both serious illness and death from COVID‐19. As reported by Diop et al., the large population of youths tends to lead to more infections in this group, but most of these infections are asymptomatic or mild and probably go undetected.^35^

This study found the highest anti‐SARS‐CoV‐2 antibody seroprevalence rate among study participants aged 10–18 years; the majority of whom did not report any clinical symptoms related to COVID‐19 in the 14 days prior to the study. This may be the results of some of the study limitations. First, the estimation of seroprevalence may be biased. We may have failed to detect newly infected individuals, or those with a weak immunologic response or low viral load,^36^ which may have led to an underestimation of the true seroprevalence rate. False positive results for the WANTAI SARS‐CoV‐2 Ab rapid test may have occurred due to cross‐reactivity from pre‐existing antibodies or other possible causes.^37^ Tso et al. found significantly higher SARS‐CoV‐2 serological cross‐reactivity in samples from SSA where specific human seasonal coronaviruses may have induced partially protective responses against SARS‐CoV‐2, compared with samples from the United States.^38^ Our seroprevalence estimation may have been overestimated due to this cross‐reactivity. However, the WANTAI SARS‐CoV‐2 Ab ELISA is recommended by WHO and has proven accurate in prior studies. An assessment of the clinical performance of four immunoassays for antibodies to SARS‐CoV‐2 in a real‐life routine care setting, conducted by Marlet et al., recommended first‐line serology testing for SARS‐CoV‐2 antibodies using WANTAI Ab because of its specificity and sensitivity that ranges from 98% to 100% and 98.0% to 99.1%, respectively, 14 days after the onset of COVID‐19 symptoms.^39^ A further limitation of our study is that we used a test that provides information regarding the presence of anti‐SARS‐CoV‐2 total antibodies, without discriminating IgG and IgM, their titration or the virus variant. It does not provide any data on who was at the acute phase of infection and who was in a later phase. Antibodies to SARS‐CoV‐2 are generally detectable in the blood several days after the initial infection, but the length of time the antibodies persist post‐infection is not yet well known.^39^ Finally, this study was limited to urban areas of Burkina Faso, and the assessment of past exposure using a questionnaire may have led to some recall bias.

The strengths of this study include the use of a rigorous methodology recommended by the WHO. This methodology was further strengthened by (1) estimating the sample size of the study based on the age and sex distribution of the population in the households and (2) performing a census in the EAs randomly selected for the study, to update the estimation of the residents before randomly sampling them for the data collection.

The high anti‐SARS‐CoV‐2 antibodies seroprevalence rate reported in our study could lead to the abandonment of COVID‐19 mitigation measures in Burkina Faso, because people may think that we have reached supposed collective immunity. However, an increase in population immunity could create selective pressure, which would favour variants that will infect people who have been immunized.^40^

## 5 CONCLUSION

This study provides strong evidence of the rapid spread of infection among city dwellers in Burkina Faso and probably throughout similar SSA countries and suggests that previous reports may have substantially underestimated the spread of infection in the region. Further studies are needed to estimate the seroprevalence of anti‐ SARS‐CoV‐2 antibodies in rural areas, where almost three quarters of the country's population lives and to elucidate the factors protecting some infected individuals from developing clinical COVID‐19. Immunization against SARS‐CoV‐2 should remain a priority for health authorities worldwide. This is particularly important in Burkina Faso because we do not have an estimation of the anti‐SARS‐CoV‐2 seroprevalence in rural areas, where almost three quarters of the country's population lives.

## AUTHOR CONTRIBUTIONS

Samiratou Ouedraogo, Isidore Tiandiogo Traoré, Dramane Kania, Blahima Konaté, Rachel Médah, Gilles Paradis, Halidou Tinto and Tienhan Sandrine Dabakuyo‐Yonli conceived the study design. Samiratou Ouedraogo, Isidore Tiandiogo Traoré, Dramane Kania, Nongodo Firmin Kaboré, Hermann Badolo, Mimbouré Yara, Blahima Konaté, Rachel Médah, Ariane Kamga Mamguem, Gilles Paradis, Halidou Tinto and Tienhan Sandrine Dabakuyo‐Yonli participated in the data collection, literature search and the preparation of the first draft of the paper. All authors participated in the writing of the manuscript, the analysis and interpretation of the results. All authors read and approved the final version of this manuscript.

## CONFLICT OF INTEREST STATEMENT

No conflict of interest declared.

### PEER REVIEW

The peer review history for this article is available at https://www.webofscience.com/api/gateway/wos/peer-review/10.1111/irv.13216.

## ETHICS STATEMENT

The study was approved by the Ministry of Health of Burkina Faso (Ref No. 2020‐00952/MS/CAB/INSP/CM) and the Burkina Faso National Ethics Committee for health research (Ref No. 2020‐8‐140).

## STATEMENT OF PATIENT CONSENT

All data collected was confidential and written informed consent was obtained from all participants prior to participation.

## Data Availability

The data presented in the manuscript are available on request.
